# Common Metabolites in Two Different Hypertensive Mouse Models: A Serum and Urine Metabolome Study

**DOI:** 10.3390/biom11091387

**Published:** 2021-09-21

**Authors:** Gaurav Baranwal, Rachel Pilla, Bethany L. Goodlett, Aja K. Coleman, Cristina M. Arenaz, Arul Jayaraman, Joseph M. Rutkowski, Robert C. Alaniz, Brett M. Mitchell

**Affiliations:** 1 Department of Medical Physiology, College of Medicine, Texas A&M University, Bryan, TX 77847, USA; gauravbaranwal@tamu.edu (G.B.); bethany.goodlett@tamu.edu (B.L.G.); coleman123@tamu.edu (A.K.C.); carenaz3@tamu.edu (C.M.A.); rutkowski@tamu.edu (J.M.R.); 2 Department of Small Animal Clinical Science, College of Veterinary Medicine & Biomedical Science, Texas A&M University, College Station, TX 77843, USA; rpilla@cvm.tamu.edu; 3 Department of Microbial Pathogenesis and Immunology, College of Medicine, Texas A&M University, Bryan, TX 77847, USA; arulj@mail.che.tamu.edu (A.J.); robert_alaniz@tamu.edu (R.C.A.); 4 Department of Biomedical Engineering, Texas A&M University, College Station, TX 77843, USA; 5 Artie McFerrin Department of Chemical Engineering, Texas A&M University, College Station, TX 77843, USA

**Keywords:** microbial metabolites, angiotensin II, salt, hypertension, metabolomics

## Abstract

Recent metabolomics studies have identified a wide array of microbial metabolites and metabolite pathways that are significantly altered in hypertension. However, whether these metabolites play an active role in pathogenesis of hypertension or are altered because of this has yet to be determined. In the current study, we hypothesized that metabolite changes common between hypertension models may unify hypertension’s pathophysiology with respect to metabolites. We utilized two common mouse models of experimental hypertension: L-arginine methyl ester hydrochloride (L-NAME)/high-salt-diet-induced hypertension (LSHTN) and angiotensin II induced hypertension (AHTN). To identify common metabolites that were altered across both models, we performed untargeted global metabolomics analysis in serum and urine and the resulting data were analyzed using MetaboAnalyst software and compared to control mice. A total of 41 serum metabolites were identified as being significantly altered in any hypertensive model compared to the controls. Of these compounds, 14 were commonly changed in both hypertensive groups, with 4 significantly increased and 10 significantly decreased. In the urine, six metabolites were significantly altered in any hypertensive group with respect to the control; however, none of them were common between the hypertensive groups. These findings demonstrate that a modest, but potentially important, number of serum metabolites are commonly altered between experimental hypertension models. Further studies of the newly identified metabolites from this untargeted metabolomics analysis may lead to a greater understanding of the association between gut dysbiosis and hypertension.

## 1. Introduction

Hypertension is a major global health issue. In the USA alone, almost one out of every two individuals develop hypertension [1]. Recent reports have identified the gut microbiome and its metabolites as important factors governing the pathophysiology of many diseases. Hypertension is one such disease, where understanding the variation in the microbiome across different experimental models, as well as in humans with hypertension, has revealed that dysbiosis is associated with disease progression [2,3]. One of the primary mechanisms by which the body’s microbiome potentially influences hypertension is through its production of unique, biologically active metabolites that target various blood-pressure-regulating organs [4,5,6].

Metabolites act as a key means of communication between the host and the microbes from which the metabolites are derived. One major class of metabolites, short-chain fatty acids (SCFA), has been repeatedly reported to play an active role in modulating cardiovascular and renal disease [6,7,8]. SCFA have been reported to reduce blood pressure by interacting with Olfr78 [9] and regulate immune cell responses through their interaction with various classes of G protein receptors [10]. A recent report has also added the effect of microbiota and its metabolite SCFAs on G protein estrogen receptor 1 (*Gper1*), which has been identified to strongly regulate blood pressure [11]. Another class of metabolites, tryptophan metabolites, were found to be elevated in patients with severe cardiovascular disease and this was associated with mortality; they were also reported to have adverse effects on the progression of chronic kidney disease [12,13,14,15]. Trimethylamine (TMA) and its metabolite trimethylamine-N oxide (TMAO) have been reported to cause endothelial dysfunction as well as hypertension and atherosclerosis [5,14,16,17,18,19]. Even though major studies have been carried out to understand the role of specific metabolites in chronic disease progression, recent advances in metabolomics approaches have opened gateways for a better identification of the metabolites associated with chronic progression of disease pathology.

Additional hypertension studies are needed to address the direct association of microbial metabolites with disease progression. A recent study by Cheema and Pluznick reported a wide array of metabolites that are increased and decreased in angiotensin-II-induced hypertension (AHTN) [20]. A key finding of the study was that metabolites that are differentially regulated in AHTN are dependent on the gut microbiota [20]. The current state of understanding of how various metabolites modulate blood pressure was recently summarized by Chakraborty et al. [21].

Given these recently identified microbial metabolites and pathways that are significantly altered in hypertension, it has yet to be determined whether these metabolites play an active role in the pathogenesis of hypertension or are altered as a result. In the current study, we performed untargeted global metabolomics analysis on serum and urine samples to evaluate common metabolites that are altered between two hypertensive models when compared to control mice. To achieve this, we examined L-NAME/high-salt-diet-induced hypertension (LSHTN) and AHTN models of hypertension in mice. These models represent different mechanisms of hypertension, as one is a low-renin model and the other is a high-renin model, and thus allow the common effect of hypertension to identify the metabolites that are altered in both. We hypothesized that a selected class of metabolites would be altered in both models, irrespective of the treatment modality used to induce hypertension, thus demonstrating a strong association between metabolite changes and hypertension. 

## 2. Materials and Methods

### 2.1. Mice

C57BL/6J, wild-type male mice were purchased from Jackson Laboratories (Bar Harbor, ME, USA). The animal use protocol TAMU #2019-0144 was approved by the Texas A&M University Institutional Animal Care and Use Committee and procedures were performed in accordance with the NIH Guidelines for the Care and Use of Laboratory Animals. All mice were between the ages of 10–17 weeks of age and weighed ~25–30 g each.

### 2.2. L-Arginine Methyl Ester Hydrochloride (L-NAME)/High Salt Diet Induced Hypertension (LSHTN)

Mice aged 10 weeks (*n* = 3), were made hypertensive by providing L-NAME (0.5 mg/mL; Sigma, St. Louis, MO, USA) in their drinking water. After 2 weeks of L-NAME treatment, all mice received regular water for 2 weeks (wash out), followed by a high-salt diet containing 4% NaCl for 3 weeks, as described previously [22,23]. Systolic blood pressure was determined using the tail-cuff method after acclimatization and training, as described previously [24]. The control mice were fed regular chow (Teklad, 8604) during the entire study period (with NaCl content of roughly 0.5%).

### 2.3. Angiotensin II Induced Hypertension (AHTN)

Mice aged 10 weeks (*n* = 3), were anesthetized with isoflurane, and subcutaneously implanted with osmotic pumps (Alzet, model 1004, Cupertino, CA, USA) filled with angiotensin II (490 ng/kg/min; BACHEM, Torrance, CA, USA). Infusions lasted 3 weeks, during which the mice had free access to normal chow and drinking water. Systolic blood pressure was determined using the tail-cuff method after acclimatization and training, as described previously [24]. The mice were fed regular chow (Teklad, 8604) during the entire study period (with NaCl content of roughly 0.5%).

### 2.4. Blood and Urine Collection

At the end of the study period, mice were placed in diuresis cages and allowed to acclimatize for 24 h, after which urine was collected from individual mice over a period of 24 h. Mice were euthanized by axillary vein exsanguination under 5% inhalational isoflurane anesthesia, with death confirmed by cervical dislocation before tissue collection. Blood samples were kept on ice and were then centrifuged at 6000× *g* for 6 min to separate serum. Serum and urine samples were stored at −80 °C until analysis.

### 2.5. Metabolite Extraction/Sample Extraction

Samples were weighed and extracted with a methanol extraction method, as described previously [25,26]. In brief, 200 µL of ice-cold methanol was added to 50 µL of serum or urine. Samples were vortexed and centrifuged to remove pelleted material. The supernatant was collected and stored at −80 °C until analysis. 

### 2.6. Mass Spectrometry Analysis

Untargeted liquid chromatography high-resolution accurate mass spectrometry (LC-HRAM) analysis was carried out at the Integrated Metabolomics Analysis Core facility at Texas A&M University on a Q Exactive Plus Orbitrap mass spectrometer (Thermo Scientific, Waltham, MA, USA) coupled with a binary pump HPLC (UltiMate 3000, Thermo Scientific Inc., San Jose, CA, USA). Full MS spectra were obtained in the positive mode at 70,000 resolution (200 m/z) with a scan range of 50–750 m/z. Full MS, followed by data-dependent scans, were obtained at 35,000 resolution (MS1) and 17,500 resolution (MS2) with a 1.5 m/z isolation window and a stepped NCE (20, 40, 60). Samples were maintained at 4 °C before injection. The injection volume was 10 µL. Chromatographic separation was achieved on a Synergi Fusion 4 µm, 150 mm × 2 mm reverse phase column (Phenomenex, Torrance, CA, USA) maintained at 30 °C using a solvent gradient method. Solvent A was water (0.1% formic acid). Solvent B was methanol (0.1% formic acid). The gradient method used was 0–5 min (10% B to 40% B), 5–7 min (40% B to 95% B), 7–9 min (95% B), 9–9.1 min (95% B to 10% B), 9.1–13 min (10% B). The flow rate was 0.4 mL/min. Sample acquisition was performed using Xcalibur software (version 4.0, Thermo Scientific Inc., San Jose, CA, USA). Data analysis was performed with Compound Discoverer 3.1 (Thermo Fisher Scientific, San Jose, CA, USA).

### 2.7. Metabolomics Data Analysis and Statistics

The analysis of metabolites was performed as described previously [27]. The data table was filtered by deleting metabolites of unknown identity. The data table containing peak intensities was uploaded to MetaboAnalyst 4.0 (https://www.metaboanalyst.ca/) (accessed on 31 July 2020), followed by log transformation and Pareto scaling for normalization of the data. From the same table, fold changes were calculated by comparing the mean of log transformed peak intensity values with respect to the control (i.e., LSHTN or AHTN/Control) using a mathematical formula (mean of LSHTN or AHTN/mean of control). The ‘*p*’ values were reported as generated from the ANOVA post hoc results, for the comparison of individual groups and the control. A metabolite was ‘significantly altered’ based on fold change >2 and ‘*p*’ value < 0.05.

Univariate analysis for all three datasets (Control, LSHTN, and AHTN) were compared with one-way ANOVA, followed by Tukey’s multiple comparison test (Control vs. LSHTN; Control vs. AHTN; LSHTN vs. AHTN). Multivariate analyses were performed within MetaboAnalyst and included PCA. Hierarchical clustering was performed with the hclust function in package stat, and the results were represented in the form of a dendrogram. Based on the ranking of features, a heatmap of all metabolites identified by ANOVA as being significantly different between groups was generated. Cutoffs based on rank were chosen over a specific significance threshold, since different statistical approaches will yield different absolute scores of significances, but most of the top-ranked features are expected to be consistent. For a heatmap, the top 50 features subjectively provide the ability to visualize both the trends and the variability of the features across samples. 

## 3. Results

**Figure 1:**
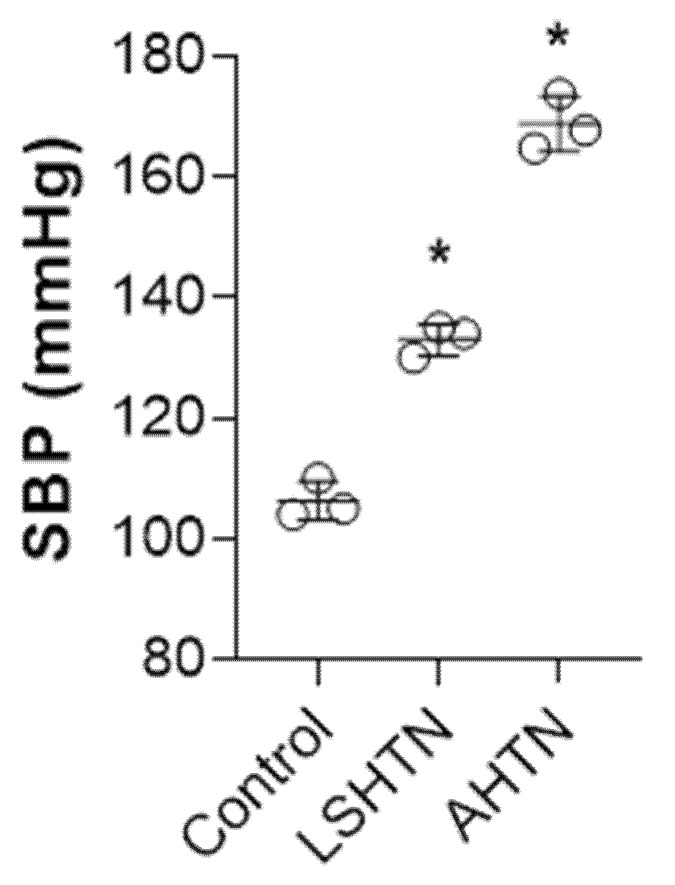
Systemic blood pressures (SBP) of control mice and mice with L-NAME/high-salt-diet-induced hypertension (LSHTN) or angiotensin II induced hypertension (AHTN). * indicates *p* < 0.05 by ANOVA, (*n* = 3).

**Figure 2:**
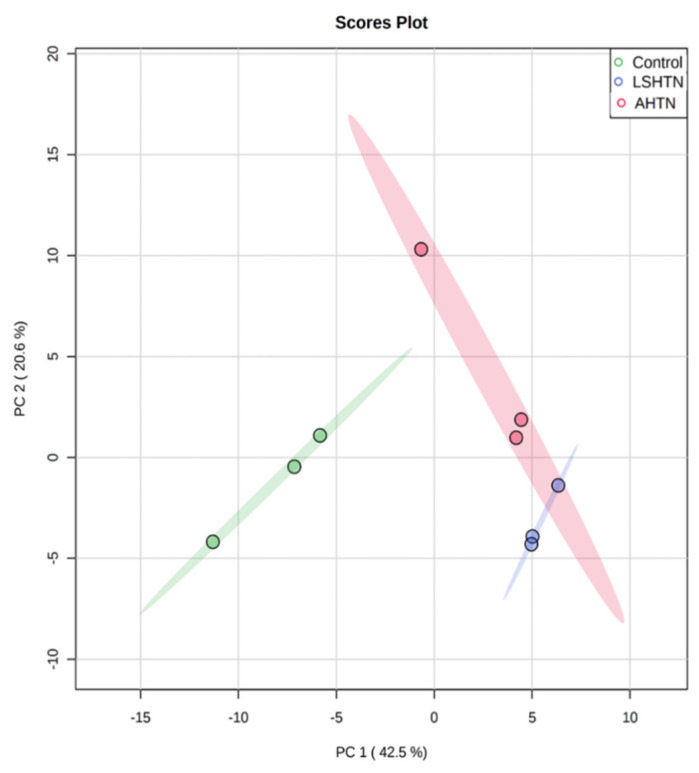
Metabolomics data of serum showing segregation of hypertensive groups from control. Principal components analysis (PCA) plot of metabolites present in the serum based on untargeted metabolomics demonstrated visible segregation based on peak intensity values of metabolites in both LSHTN and AHTN mice when compared to control mice, (*n* = 3).

To identify common metabolites across different models of hypertension, mice with LSHTN and AHTN were generated. There were no mortalities during the study. At termination, the average systolic blood pressure was 133 ± 2.6 mm Hg for LSHTN mice and 167 ± 3.5 mm Hg for AHTN mice (Figure 1). At the end of the respective study, serum and urine samples were collected from each mouse and metabolomics were performed. Based on the metabolomics data, we detected the presence of 607 metabolites in the serum and 652 metabolites in the urine. The heatmaps of the top 50 metabolites in the serum and urine for all groups are depicted in Supplementary Data Figures S1 and S2, respectively. Principal components analysis (PCA) of serum metabolic data demonstrated a clear segregation of hypertensive group samples from control samples (Figure 2). Both hypertensive groups had an overlap, suggesting that hypertension per se is an important factor determining the overall metabolite profile (control vs. LSHTN/AHTN).

### 3.1. Significantly Altered Metabolites in the Serum of Hypertensive Mice

The primary objective of this study was to identify the common metabolites that are significantly altered in different mouse models of hypertension. We found 41 metabolites to be significantly altered across the groups based on an ANOVA for each metabolite. Of the 41 metabolites that were significantly altered across any hypertensive group, four metabolites were increased, and 10 metabolites were uniformly decreased in both hypertensive models when compared to controls. The remaining serum metabolites only significantly altered in LSHTN or AHTN models when compared with controls (Supplementary Materials Tables S1 and S2, respectively). No metabolites were identified that were significantly changed in opposing directions across the models. 

**Figure 3:**
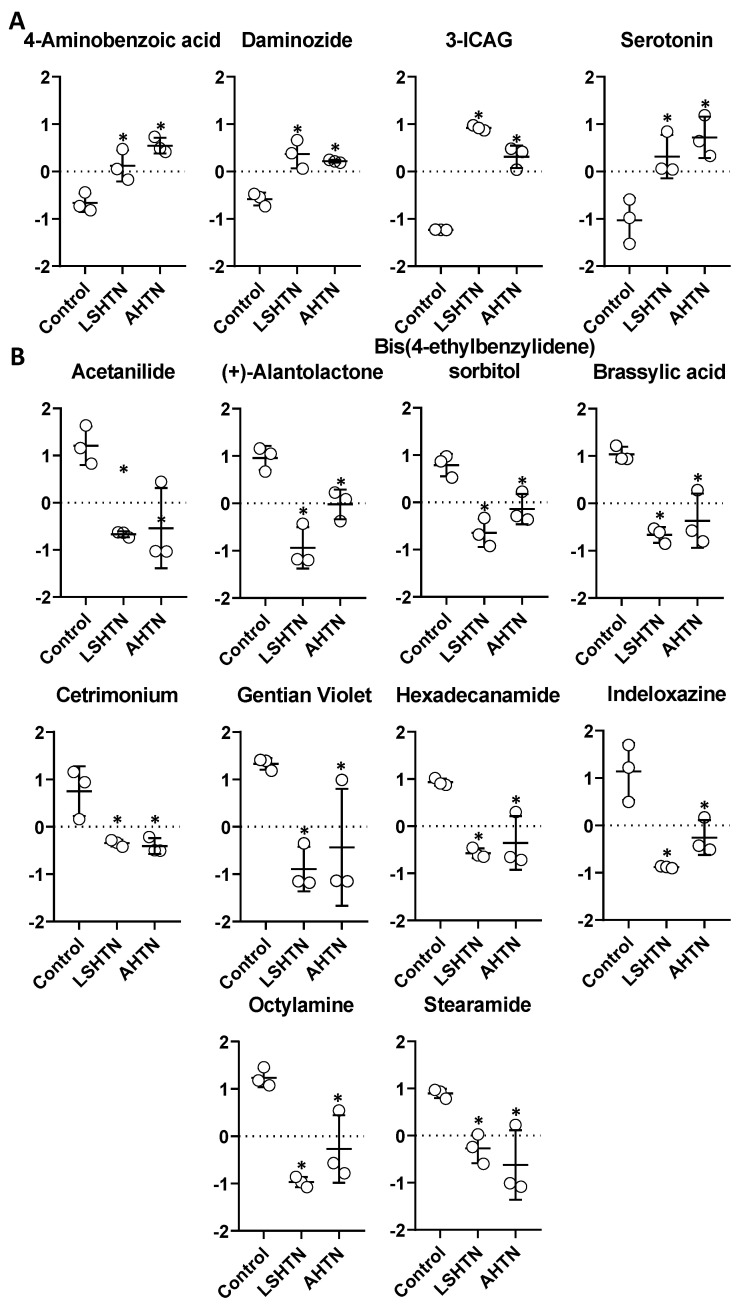
Common serum metabolites increased or decreased significantly in both LSHTN and AHTN mice compared to control mice. Serum metabolites that were significantly increased in both hypertensive groups are shown in (**A**). Serum metabolites that were significantly decreased in both hypertensive groups are shown in (**B**). * indicates *p* < 0.05 by one-way ANOVA. Data are plotted as fold changes in log transformed values of the normalized peak intensity values, (*n* = 3).

The four metabolites that were significantly increased in both hypertensive groups are depicted in Figure 3A. Aminobenzoic Acid was increased 1.6-fold in LSHTN and 1.9-fold in AHTN; Daminozide was increased 1.7-fold and 1.6-fold in LSHTN and AHTN, respectively; 3-Indole Carboxylic Acid Glucuranide (ICAG) was increased 3.8-fold in LSHTN and 3-fold in AHTN; Serotonin was increased 2.4-fold in LSHTN and 2.8-fold in AHTN. There was a significantly higher level of ICAG in LSHTN vs. AHTN, indicating an added impact of a high-salt diet on this metabolite. The levels of these four metabolites were negligible in serum samples from control mice when compared to the hypertensive mice, suggesting that these metabolites are universally elevated during hypertension.

### 3.2. Serum Metabolites Decreased in Hypertensive Mice

The 10 metabolites that were significantly decreased in both hypertensive models are depicted in Figure 3B. Acetanilide was decreased 2.4-fold in LSHTN and 2.2-fold in AHTN; (+)-Alantolactone was decreased 2.8-fold in LSHTN and 1.5-fold in AHTN; Bis (4-ethylbenzylidene) sorbitol was decreased 2-fold in LSHTN and 1.5-fold in AHTN; Brassylic Acid was decreased 2.3-fold in LSHTN and 1.9-fold in AHTN; Cetrimonium was decreased 1.7-fold in both LSHTN and AHTN; Gentian Violet was decreased 3-fold in LSHTN and 2.1-fold in AHTN; Hexadecanamide was decreased 2-fold in LSHTN and 1.8-fold in AHTN; Indeloxazine was decreased 2.8-fold in SSHTN and 1.8-fold in AHTN; Octylamine was decreased 3.1-fold in LSHTN and 1.9-fold in AHTN; Stearamide was decreased 1.6-fold in LSHTN and 2.1-fold in AHTN. (+)-Alantolactone was significantly decreased in LSHTN mice compared to AHTN mice, which may be due to the high-salt diet. The other nine decreased metabolites were similar between the hypertensive groups, suggesting that the means of inducing hypertension was not a factor. 

### 3.3. Minimal Impact on Urine Metabolites across Hypertensive Models 

**Figure 4:**
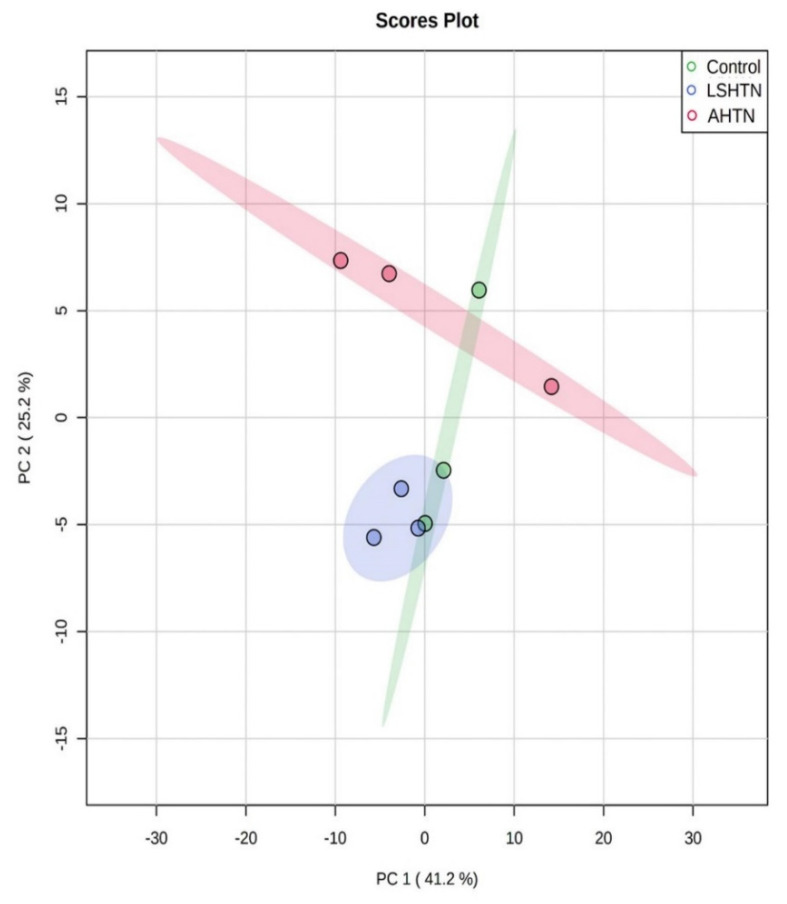
Metabolomics data of urine showing intermixed results of the hypertensive groups with the control group. PCA plot of metabolites present in the urine based on untargeted metabolomics demonstrated no visible segregation among metabolites of LSHTN and AHTN mice when compared to control mice, (*n* = 3).

Metabolomics analysis of the urine collected from these mouse models of hypertension identified that there is much greater variability in urine metabolites, as demonstrated in the PCA plots (Figure 4), potentially complicating comparison analysis. Based on the heatmap, we observed a few clusters that were unique to LSHTN or AHTN, but there were no metabolites that were universally altered across hypertensive groups (Supplementary Materials Figure S2).

## 4. Discussion

Understanding the role of the body’s metabolites is an emerging approach to unravel some potential pathogenic mechanisms in hypertension. Whether changes in metabolites are causative, and potentially therapeutic targets, or are biomarkers of disease remains to be fully identified. In the current study, we identified the metabolites that were significantly altered across two different mouse models of hypertension, LSHTN and AHTN, through an untargeted metabolomics approach in serum and urine. We found 41 metabolites in the serum and 6 metabolites in the urine to be significantly altered in either hypertensive group. Importantly, 4 serum metabolites were increased significantly, and 10 serum metabolites were decreased significantly across both hypertensive groups, one a low-renin model and the other a high-renin model, indicating that these may be important in the pathology of hypertension. Recently published work has identified a potentially strong role for altered metabolites in hypertension [3,13,26,27,28,29,30,31,32]. Cheema and Pluznick performed a well-controlled study comparing germ-free and conventionally housed AHTN mice [20]. They identified 4 metabolites that were increased significantly and 8 that were decreased significantly, with the identified pathways including benzoate, tyrosine, and tryptophan metabolism. With no changes observed in germ-free mice, metabolome changes could, therefore, be attributed to an altered gut microbiota during AHTN. While none of the specific metabolites that we identified in the current study were shared with Cheema and Pluznick’s study, pathway analysis may suggest common changes in classes, such as tryptophan metabolites with carboxylic acid glucuronide, a tryptophan metabolite, being found increased in the current study. Again, whether these specific metabolites, classes, or pathways play a role in the pathogenesis of hypertension, or merely result from it, remains to be studied.

The 14 metabolites identified in our study were cross-referenced with 17 hypertensive metabolome studies previously published in either humans or animals. One such study was Tian et al., in which the investigators utilized ultra-high-performance liquid chromatography coupled with Q Exactive hybrid quadrupole-Orbitrap mass spectrometry to compare SHR and WKY serum metabolites [28]. This group also found that serotonin is increased significantly in hypertension compared to the control [28]. Serotonin receptors play a well-documented role in the pathogenesis of hypertension; therefore, it is not surprising that serotonin, a tryptophan metabolite, increases in hypertensive models [29,33]. Another study comparing plasma from pulmonary arterial hypertensive patients to a control group found that stearamide, a fatty acid, has decreased expression [30]. While that study is not primarily about essential hypertension, it agrees with our finding that stearamide expression is decreased in two mouse models of hypertension. Of the 14 compounds identified as significantly altered in our study, however, serotonin was the only one that was directly identified in the 17 hypertensive metabolome studies.

The metabolites hexadecanamide, a fatty amide, and ICAG, a flavonoid, were either not found in the 17 papers or were found to be expressed differently than in the current study. Hexadecanamide was only found to be significantly changed in one study, in which the serum of healthy and hypertensive individuals underwent GC/MS and HPLC/MS to find biomarkers of hypertension, but the study found that hexadecanamide was increased, whereas our findings identified a significant decrease in both hypertensive models [30]. The differences between the concentration of hexadecanamide in the serum is interesting and suggests that future studies should be carried out to clarify its role in hypertension. While hexadecanamide was only found in one study, an increase in hexadecanoic acid, a fatty acid, was determined in several of the 17 hypertensive studies [30,34,35]. Hexadecanoic acid is the most common saturated fatty acid in the human body and a disruption of its ratio in membrane fluidity has been tied to a variety of physiological diseases, such as diabetes, cardiovascular disease, and hypertension [36]. Hexadecanamide is directly derived from hexadecanoic acid [31], but their combined role in hypertension is not fully understood. Another study cited an increase in hexadecenoic acid, which is a hexadecenoic acid containing a trans-double bond at the 2 position [37]. Hexadecenoic acid has a role in the de novo lipogenesis pathway and heart failure, however more research is needed to define its direct role in hypertension [38]. Similarly, several studies cited an increase in or aberrant changes in stearic acid, a carboxylic acid derivative of stearamide, in hypertension [2,35,39]. While stearamide does not have a reported role in essential hypertension, stearic acid is inversely associated with diastolic blood pressure and may be an indication of coronary heart disease risk [40]. How stearic acid impacts stearamide in hypertension has yet to be reported. Similarly, while ICAG was not specifically referenced, several forms of indole were reported to significantly change in various hypertensive models, but the derivative form of indole and its regulation differed greatly across studies [2,41,42,43]. Indole has reported effects on arterial blood pressure and may be a mediator between the circulatory system and gut bacteria [44]. More research will need to be performed to elucidate the role of ICAG in hypertension. 

None of the 17 studies listed Aminobenzoic acid, Daminozide, Acetanilide, (+)-Alantolacetone, Bis (4-ethylbenzylidene) sorbitol, Brassylic acid, Cetrimonium, Gentian violet, Ideloxazine, or Octylamine as significantly altered metabolites in hypertension, nor were any of their derivatives found. Although it was not cited, aminobenzoic acid may be linked to hypertension through one of its derivatives, Aminaftone, which suppresses the effects of pulmonary hypertension by downregulating endothelin-1 receptors (37). Hexadecanamide and brassylic acid are long-chain fatty acid derivatives, and little is known of their biological activity. It is confounding that (+)-Alantolacetone, Bis (4-ethylbenzylidene) sorbitol, daminozide, and gentian violet were identified in our study, as diet, water and drug could not explain their shared changes across the hypertensive models. Similarly, none of the published literature has suggested any direct association of Acetanilide, Octylamine, Brassylic Acid, Indeloxazine, Hexadecanamide, Bis (4-ethylbenzylidene) sorbitol, and (+)-Alantolactone with hypertension. This suggests that these metabolites could be participating in an unknown pathway that has yet to be elucidated. Further research is needed to clarify their roles in metabolism and hypertension. 

The current study identified no shared significant changes in urinary metabolites across the two models. This could be, in part, due to the method of urine collection, not using refrigeration or preservative, or it could be due to the limited number of mice in the study. That 14 significantly changed metabolites were identified in the serum, however, support that these findings and these compounds likely have relevancy to hypertension. Increasing the number of samples may identify additional compounds and pathways shared across models of hypertension, reflected, potentially, in the blood, urine, and feces of mice. Another limitation of the current study is that the data were not distributed normally due to the small number of samples. However, the PCA plots show that the differences are dramatic enough to be visible even with small numbers, and future studies with more samples will confirm these results. Lastly, other limitations of this study were the age difference between the LSHTN and AHTN groups, which was four weeks at the time of termination, as well as the level of blood pressure between the two hypertensive groups, which may also have had an impact on the overall outcome of the study.

In conclusion, the serum and urine metabolites of two different mouse models of hypertension were studied using an untargeted metabolomics approach, and 14 serum metabolites were identified to be shared across hypertensive groups. Metabolites have the potential to interact with a series of signaling pathways relevant to blood pressure regulation. Those common to independent models of hypertension have an increased potential to serve as biomarkers of hypertension or targets in the treatment of hypertension. Further studies are needed to better understand how these metabolites mechanistically affect the pathophysiology of hypertension. 

## Data Availability

The data presented in this study are openly available in FigShare at 10.6084/m9.figshare.16637860.

## Supplementary Materials

The following are available online at https://www.mdpi.com/article/10.3390/biom11091387/s1, Figure S1: Heatmap showing clustered average of top 50 metabolites in serum samples from Control, LSHTN, and AHTN mice; Figure S2: Heatmap showing clustered average of top 50 metabolites in urine samples from Control, LSHTN, and AHTN mice; Table S1: Serum metabolites significantly altered in LSHTN mice compared to control mice; Table S2: Serum metabolites significantly altered in AHTN mice compared to control mice.
