# Supplementary material for: Common Metabolites in Two Different Hypertensive Mouse Models: A Serum and Urine Metabolome Study
Source: Biomolecules. 2021 Sep 21;11(9):1387. doi: 10.3390/biom11091387 (PMC8467937; doi:10.3390/biom11091387)
Supplement: Supplementary file 1 [file biomolecules-11-01387-s001.zip › biomolecules-1371949-supplementary.pdf]

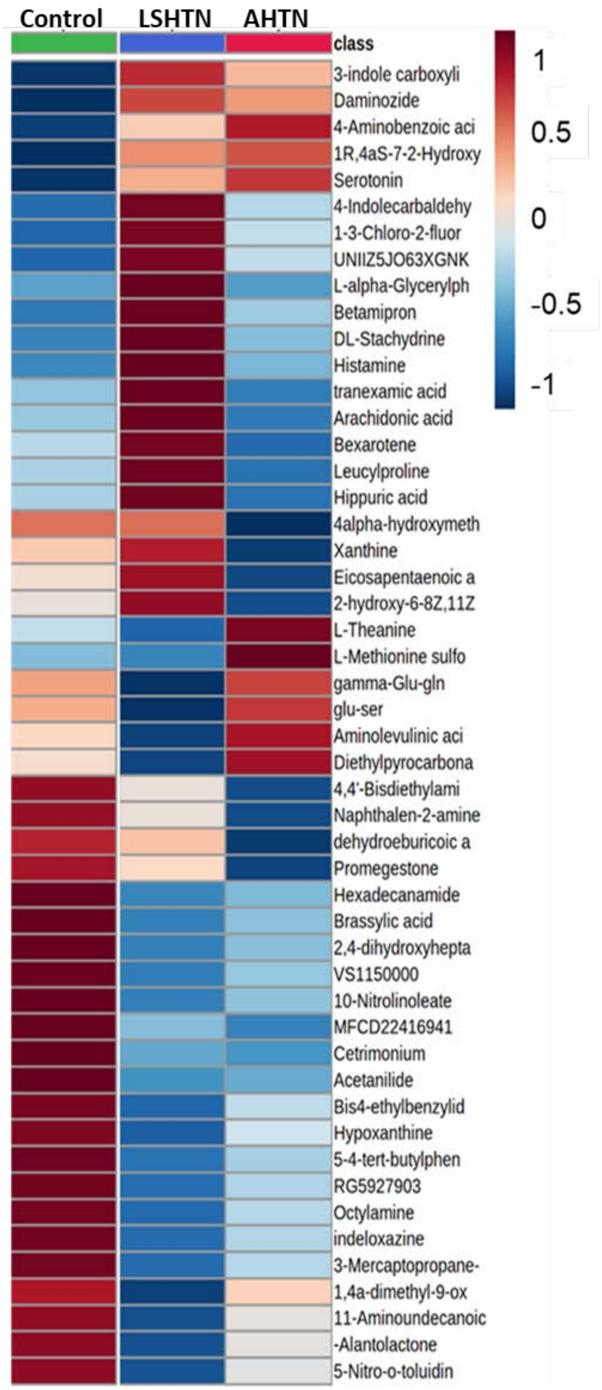

Supplementary Figure S1: Heatmap showing clustered average of top 50 metabolites in serum samples from Control, LSHTN, and AHTN mice.

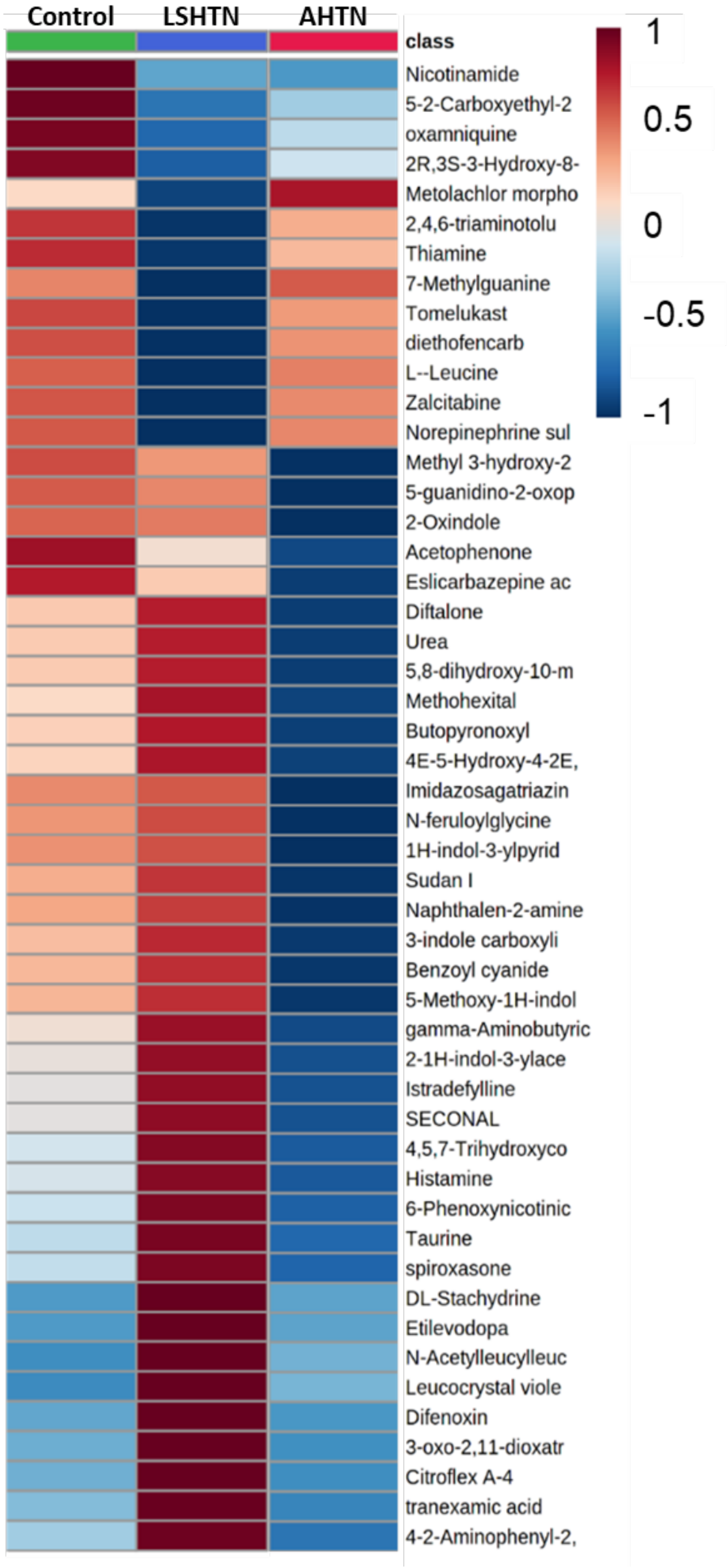

Supplementary Figure S2: Heatmap showing clustered average of top 50 metabolites in urine samples from Control, LSHTN, and AHTN mice.

Supplementary Table S1: Serum metabolites significantly altered in LSHTN mice compared to control mice.

Serum metabolites significantly altered in control vs. LSHTN	Fold Change	*p* value
1-3-Chloro-2-fluorophenyl-3-dimethylaminoprop-2-en-1-one	2.5	0.003
2-hydroxy-6-8Z,11Z-pentadeca-8,11,14-trien-1-ylbenzoic acid	2.3	0.001
4-Indolecarbaldehyde	2.6	0.001
Bexarotene	2.5	0.003
DL-Stachydrine	3.1	<0.001
DL-Tryptophan	2	0.002
Eicosapentaenoic acid	2.3	0.001
Hippuric acid	2.4	0.002
Indole-3-lactic acid	2.4	0.007
L-alpha-Glycerylphosphorylcholine	2.5	<0.001
Leucylproline	2.5	<0.001
NP-020078	2.9	0.002
tranexamic acid	2.6	<0.001
trans-3-Indoleacrylic acid	2.5	0.004
Tryptophan degradation	3.2	0.007
Uric acid	2.7	0.002

Supplementary Table S2: Serum metabolites significantly altered in AHTN mice compared to control mice.

**Serum metabolites significantly altered in control vs. AHTN**	**Fold Change**	***p* value**
3'-Hydroxycotinine	2	0.005
L-Methionine sulfoxide	2.5	0.006
